# Early-Life Exposure to Polycyclic Aromatic Hydrocarbons and ADHD Behavior Problems

**DOI:** 10.1371/journal.pone.0111670

**Published:** 2014-11-05

**Authors:** Frederica P. Perera, Hsin-wen Chang, Deliang Tang, Emily L. Roen, Julie Herbstman, Amy Margolis, Tzu-Jung Huang, Rachel L. Miller, Shuang Wang, Virginia Rauh

**Affiliations:** 1 Department of Environmental Health Sciences, Mailman School of Public Health, Columbia University, New York, New York, United States of America; 2 Columbia Center for Children's Environmental Health, Columbia University, New York, New York, United States of America; 3 Department of Biostatistics, Mailman School of Public Health, Columbia University, New York, New York, United States of America; 4 Division of Child & Adolescent Psychiatry and the Center for Developmental Neuropsychiatry, Department of Psychiatry, the New York State Psychiatric Institute and the College of Physicians and Surgeons, Columbia University, New York, New York, United States of America; 5 Division of Pulmonary, Allergy and Critical Care of Medicine, Department of Medicine, College of Physicians and Surgeons, Columbia University, New York, New York, United States of America; 6 Division of Pediatric Allergy and Immunology, Department of Pediatrics, College of Physicians and Surgeons, Columbia University, New York, New York, United States of America; 7 The Heilbrunn Department of Population and Family Health, Columbia University, New York, New York, United States of America; Oregon State University, United States of America

## Abstract

**Importance:**

Polycyclic aromatic hydrocarbons are widespread urban air pollutants from combustion of fossil fuel and other organic material shown previously to be neurotoxic.

**Objective:**

In a prospective cohort study, we evaluated the relationship between Attention Deficit Hyperactivity Disorder behavior problems and prenatal polycyclic aromatic hydrocarbon exposure, adjusting for postnatal exposure.

**Materials and Methods:**

Children of nonsmoking African-American and Dominican women in New York City were followed from *in utero* to 9 years. Prenatal polycyclic aromatic hydrocarbon exposure was estimated by levels of polycyclic aromatic hydrocarbon- DNA adducts in maternal and cord blood collected at delivery. Postnatal exposure was estimated by the concentration of urinary polycyclic aromatic hydrocarbon metabolites at ages 3 or 5. Attention Deficit Hyperactivity Disorder behavior problems were assessed using the Child Behavior Checklist and the Conners Parent Rating Scale- Revised.

**Results:**

High prenatal adduct exposure, measured by elevated maternal adducts was significantly associated with all Conners Parent Rating Scale-Revised subscales when the raw scores were analyzed continuously (N = 233). After dichotomizing at the threshold for moderately to markedly atypical symptoms, high maternal adducts were significantly associated with the Conners Parent Rating Scale-Revised DSM-IV Inattentive (OR = 5.06, 95% CI [1.43, 17.93]) and DSM-IV Total (OR = 3.37, 95% CI [1.10, 10.34]) subscales. High maternal adducts were positivity associated with the DSM-oriented Attention Deficit/Hyperactivity Problems scale on the Child Behavior Checklist, albeit not significant. In the smaller sample with cord adducts, the associations between outcomes and high cord adduct exposure were not statistically significant (N = 162).

**Conclusion:**

The results suggest that exposure to polycyclic aromatic hydrocarbons encountered in New York City air may play a role in childhood Attention Deficit Hyperactivity Disorder behavior problems.

## Introduction

Polycyclic aromatic hydrocarbons (PAH), such as benzo[a]pyrene (B[a]P), are toxic air pollutants released during incomplete combustion of fossil fuel, tobacco, and other organic material [1]. They are also found in the diet. In New York City (NYC) and other urban areas, traffic and residential heating are major local sources. There is also some contribution from coal-burning sources in states upwind. Urban, minority populations in the U.S. often have disproportionate exposure to air pollution and are at greater risk for adverse health and developmental outcomes from this exposure [2]–[5]. All of the mothers in the Columbia Center for Children's Environmental Health (CCCEH) NYC cohort had detectable levels of PAH in prenatal personal air samples; 42% had detectable levels of B[a]P-DNA adducts in maternal blood; and 46% had detectable levels of B[a]P-DNA adducts in cord blood. B[a]P is considered a representative PAH and is highly correlated with other PAH class members [6]. PAH-DNA adducts reflect individual exposure to PAH, integrating exposure over a 2–3 month period [7]and via different routes (primarily inhalation and ingestion). Adducts provide a biologic dosimeter as they not only reflect inter-individual differences in exposure and uptake of PAH but also in detoxification and DNA repair [8],[9].

Because of the heightened susceptibility of the fetus and young child, exposures to PAH and other environmental pollutants during the prenatal and early postnatal stages are of particular concern [10]–[13]. During the fetal period and early childhood years, the brain is rapidly developing and vulnerable to neurotoxic insults that may manifest as adverse outcomes in childhood and adulthood [14], [15]. Laboratory studies of PAH exposure during the prenatal, neonatal, or adult periods have reported a range of neurodevelopmental and behavioral effects, [16], [17] including hyperactivity [18],[19]. In the present CCCEH cohort, prenatal exposure to PAH measured by prenatal air monitoring or B[a]P-DNA adducts in maternal or umbilical cord blood at delivery was associated with developmental delay at age 3 [20], reduced IQ at age 5 [21], and symptoms of anxiety/depression and attention problems at ages 6–7 [6].

Attention-deficit/hyperactivity disorder (ADHD) is the most common behavioral disorder diagnosed in children [22] and is often accompanied by anxiety and depression [23]–[26]. In our cohort, ADHD behavior problems and anxiety/depression at age 9 were significantly correlated (r = 0.43, p<0.0001). Children with ADHD are at increased risk of substance abuse, conduct, and mood disorders [27]–[30]. Family history, certain environmental contaminants, alcohol use, maternal smoking during pregnancy, pregnancy and delivery complications, and psychosocial adversity have been implicated or identified as risk factors for ADHD [31], [32].

Prior data on air pollution and ADHD are suggestive. For example, a cross-sectional study found an association between ambient particulate matter (PM_10_) and childhood ADHD [33]. In a longitudinal study, estimated exposure during infancy to elemental black carbon, based on air sampling data and land use regression modeling, was significantly associated with ADHD-related symptoms [34]. Another cohort study reported an association between attention and children's lifetime exposure to black carbon based on children's residence and a spatiotemporal model [35]. Ours is the first report of associations between individual measures of early-life exposure to PAH pollutants and ADHD behavior problems in children.

## Methods

### Sample selection

A complete description of the NYC cohort appears elsewhere [20], [36]. Briefly, African-American and Dominican women who resided in Washington Heights, Harlem, or the South Bronx in NYC, U.S., were recruited between 1998 and 2006 through local prenatal care clinics. Enrollment was restricted to women who were non-active cigarette smokers; ages 18–35; non-users of other tobacco products or illicit drugs; free of diabetes, hypertension, or known HIV; and who had initiated prenatal care by the 20^th^ week of pregnancy. The Institutional Review Board of the Columbia University Medical Center approved the study. Mothers signed a consent form, approved by the IRB, for themselves and their children at the time of enrollment and at every subsequent visit. The children sign an IRB-approved assent form beginning at age 7. The consent and assent forms are available in English and Spanish and clearly explain the study goals and procedures.

The sample included in the present analysis is composed of the children who had available data on at least one adduct measure (maternal or newborn), the CPRS and the CBCL assessments, and all covariates of interest (N = 250).

### Maternal/child characteristics and home caretaking environment

#### Demographic, health and environmental conditions

A 45-minute structured questionnaire was administered by a trained bilingual interviewer during the last trimester of pregnancy to obtain demographic information, residential history, and health and environmental data such as active smoking (to confirm non-active smoking status) and passive smoking [36]. The questionnaire also elicited information on dietary PAH (consumption of broiled, fried, grilled or smoked meat), and socioeconomic information related to income and education. Postnatal interviews were administered in person at 6 months and annually thereafter to determine changes in residence, exposure to environmental tobacco smoke (ETS), and health and environmental conditions.

#### Maternal demoralization

Maternal demoralization, a measure of maternal nonspecific psychological distress that has been linked to neurodevelopment [37]–[39], was measured at each visit by the Psychiatric Epidemiologic Research Instrument Demoralization Scale (PERI-D) [40].

#### Non-verbal intelligence

The Test of Non-Verbal Intelligence-Second Edition (C-TONI-2) [41] was administered to the mothers when the child was about 3 years old.

#### Home assessment

Caldwell and Bradley's Home Observation for Measurement of the Environment (HOME) [42] was administered in the home by research workers, also when the child was about 3 years old, to assess physical and interactive characteristics of the child rearing environment.

#### Maternal ADHD

At the child's 7 year visit, mothers completed the Conners Adult ADHD Rating Scales (CAARS) [43]. Given the high heritability rate of ADHD [44], maternal ADHD symptoms on the CAARS were included as a covariate in our analyses.

#### Child anxiety/depression

Childhood ADHD and anxiety/depression are frequently comorbid conditions [24]. The continuous score for symptoms of anxiety/depression on the CBCL at age 9 [45] was included as a covariate.

### Independent variables

#### Prenatal exposure: PAH-DNA adducts

Following delivery, maternal blood and umbilical cord blood samples were collected. Within several hours following collection, samples were transported to the CCCEH Molecular Epidemiology Laboratory, processed, and stored at −70°C. B[a]P–DNA adducts in extracted white blood cell DNA were analyzed using the high performance liquid chromatography (HPLC)/fluorescence method which detects B[a]P tetraols [12], [46]. Not all participants had adequate DNA quantity for adduct analysis.

#### Postnatal exposure: urinary PAH metabolites

At the CDC, a suite of PAH metabolites was measured in spot urine (collected from the child at ages 3 and 5) using automated liquid-liquid extraction and gas chromatography/isotope dilution high-resolution mass spectrometry [47]–[49]. Although PAH urinary metabolite have a short lifetime (half-life of 6–35 hours) [50], in conditions of chronic exposure they provide a useful measure of exposure to PAH [47], [48]. Specific gravity (SG) measurements were used to control for urinary dilution of the samples using the following formula: freshweight metabolites for the subject*(mean SG-1)/(SG for that subject-1) [51], [52].

### Behavioral outcomes

ADHD behavior problems were assessed using two complementary parent-report instruments: the CBCL for ages 6–18 (CBCL) [53] and the CPRS- Revised: Long Version [54]. The CBCL is a screening instrument assessing childhood competencies, adaptive functioning, and problems [45]. The CPRS is a focused assessment of childhood ADHD and its common comorbid disorders [54], [55]. Both are widely used instruments that measure ADHD problems and attention function and have been used to study their associations with diverse environmental contaminants [56]–[59]. Both instruments yield scales derived from the DSM-IV [60] that are intended to screen for ADHD-behavior problems and indicate those children requiring follow-up. Mothers self-administered the 80-item CPRS [54] and the 118-item CBCL [53] when their children were 9 years old, under the guidance of trained research workers. Outcomes analyzed included the CBCL DSM-oriented Attention Deficit/Hyperactivity Problems scale, and the CPRS ADHD Index and DSM-IV subscales (denoted as “Total”, “Inattentive”, and “Hyperactive-Impulsive”). The “Total” DSM-IV measure comprises the “Inattentive” and “Hyperactive-Impulsive” subscales. For both instruments, the child's responses were scored and summed to a raw score. T-scores were derived from raw scores based on the normative comparison sample as described in the administration manual and used to determine the child's classification [53], [54]. On the CBCL DSM-oriented Attention Deficit/Hyperactivity Problems score, children above the 93^rd^ percentile were classified as “borderline clinical”, and those below the 93^rd^ percentile were classified in the normal range [53]. The CPRS DSM-IV subscales and ADHD Index scores were dichotomized based on the classification of a T score >65 as “moderately to markedly atypical” and a T score ≤65 as “in the normal range” [54].

### Statistical Analysis

As in prior analyses [6], adduct levels were dichotomized as detectable/non-detectable (“high/low”), with detectable levels observed in 42% of maternal and 46% of cord blood samples in the whole cohort. Dichotomization of exposure variables is less vulnerable to measurement error and permits comparison of the most highly exposed children to children with lower exposure. In our analyses, (1-hydroxynaphthalene, 2-hydroxynaphthalene, 2-hydroxyfluorene, 3-hydroxyfluorene, 9-hydroxyfluorene, 1-hydroxyphenanthrene, 2-hydroxyphenanthrene, 3-hydroxyphenanthrene, 4-hydroxyphenanthrene) were summed to provide a composite measure denoted “PAH metabolites”. PAH metabolites in child urine at ages 3 or 5 were dichotomized at the respective medians for the entire cohort and treated as “high/low”. In terms of data analysis, the age 5 metabolite level was preferentially selected, but if that measure was missing, the age 3 metabolite level was used. In secondary analyses, adducts and PAH metabolites were also treated as a continuous variable after log transformation.

Covariates were selected based on whether they were significant contributors to the model (at p≤0.1) for at least one of the outcomes and included: prenatal ETS exposure, child's sex, child's ethnicity, child's gestational age, mother's intelligence, mother's completed years of education prior to birth of the child, maternal prenatal demoralization, maternal ADHD symptoms, child's exact age at assessment (in months), the quality of the early home caretaking environment, and season at time of monitoring (heating vs. non-heating) (Table 1). We further adjusted for child anxiety/depression at age 9 since it is a well-documented comorbid condition with ADHD and symptoms overlap [24]. Moreover, we have previously found associations of PAH with child anxiety/depressive symptoms [6]. Dietary PAH, measured prenatally during the third trimester, was not a predictor of outcomes at p≤0.1. The associations between the dichotomized PAH exposure variables and continuous raw scores and dichotomized T scores for ADHD-related behavior were analyzed by Poisson and logistic regression, respectively.

**Table 1:** Characteristics of the children included in the analysis and those not included due to missing data.

	Subjects included in the analysis (N = 250)^a^	Subjects not included in the analysis (N = 364)^b^	
Variables	Mean ±SD or %	Mean ±SD or %	p-value
High maternal adducts^c^	37.34%	44.25%	0.10
High cord adducts^c^	39.51%	49. 37%	0.07
High urinary PAH metabolites at ages 3 or 5^d^	49.20%	55.07%	0.22
Log-transformed urinary PAH metabolites at ages 3 or 5^e^	9.02±0.89	9.03±0.80	0.86
Log-transformed maternal adduct (per 10^8^ nucleotides)	−1.73±0.47	−1.68±0.48	0.20
Log-transformed cord adduct (per 10^8^ nucleotides)	−1.67±0.53	−1.59±0.53	0.13
CBCL DSM-oriented Attention Deficit/Hyperactivity Problems (% with borderline or clinical diagnosis)^f^	7.20%	10.66%	0.32
CPRS subscales (% categorized as moderately to markedly atypical)^g^			
ADHD Index	8.40%	7.87%	1.00
DSM-IV Inattentive	8.40%	5.51%	0.41
DSM- IV Hyperactive- Impulsive	10.80%	14.17%	0.40
DSM-IV Total	10.40%	7.87%	0.47
Prenatal ETS exposure (% yes)	33.20%	37.36%	0.30
**Child sex (% female)**	**57.60%**	**48.35%**	**0.03** *
Child ethnicity (AA%)^h^	40.00%	33.24%	0.09
Gestational age (in weeks)^i^	39.35±1.37	39.34±1.44	0.94
Maternal intelligence^j^	20.82±8.78	20.25±8.62	0.47
Maternal education (%≥ high school education)	64.80%	59.83%	0.24
Maternal demoralization score	1.15±0.61	1.18±0.67	0.67
Maternal ADHD (CAARS ADHD index raw score)^k^	38.79±8.87	37.67±7.16	0.15
CBCL age (in months)^l^	108.01±1.83	108.78±4.60	0.14
**CPRS age (in months)** l	**107.96±2.02**	**109.15±4.97**	**0.03** *
Home environment^m^	40.09±5.94	39.33±6.09	0.18
Heating season (% yes)^n^	54.0%	56.52%	0.64
Child anxiety/depression (CBCL anxiety/depression raw score)^o^	2.74±3.02	2.53±2.80	0.53

a Subjects were included if they had data for maternal adducts and/or cord adducts, as well as data on CBCL, CPRS outcome and all covariates of interest.

b Subjects not included are those that had available data on cord and/or maternal adducts but were missing data on the CBCL, CPRS outcomes and/or any covariates included in the final model.

c Adduct levels were dichotomized as detectable/non-detectable (“high/low”).

d PAH metabolites in child urine at ages 3 or 5 were dichotomized at the respective medians (“high/low”).

e Children with urinary PAH metabolite measurement at ages 3 or 5.

f Based on T score. Borderline or clinical defined as percentile ≥93^rd^.

g Based on T score. Moderately to markedly atypical defined as T-score >65.

h Percent African American; the remainder are Dominican.

i Based on medical record data.

j Nonverbal intelligence measured by the TONI-2.

k Measure of maternal ADHD.

l Age at administration.

m HOME Inventory as a measure of the home caretaking environment.

n Third trimester in heating season.

o Based on CBCL Anxious/Depressed Syndrome Scale measured at age 9.

*p-value <0.05.

## Results

Maternal and cord adducts were not significantly correlated with prenatally air monitored PAH, ETS, or dietary PAH.

Table 1 presents the socio-demographic, outcome, and exposure characteristics of the children who had available data on maternal or cord adducts, stratifying on whether or not they had data on neurobehavioral outcomes and covariates of interest and were thus included (N = 250) or not included (N = 364) in the analysis. The two groups were similar except that the group included had a higher proportion of females and were younger at the CPRS assessment, though all assessments were given at approximately age 9. The level of adducts and percentage characterized as high vs. low did not differ between those included and not included. Comparing the group included (N = 250) with those children who did not have maternal or cord adduct data (N = 111), there were differences in terms of exact age at assessment for the CBCL and CPRS, home inventory scores, and percentage of mothers that had completed high school (data not shown).

**Figure 1:**
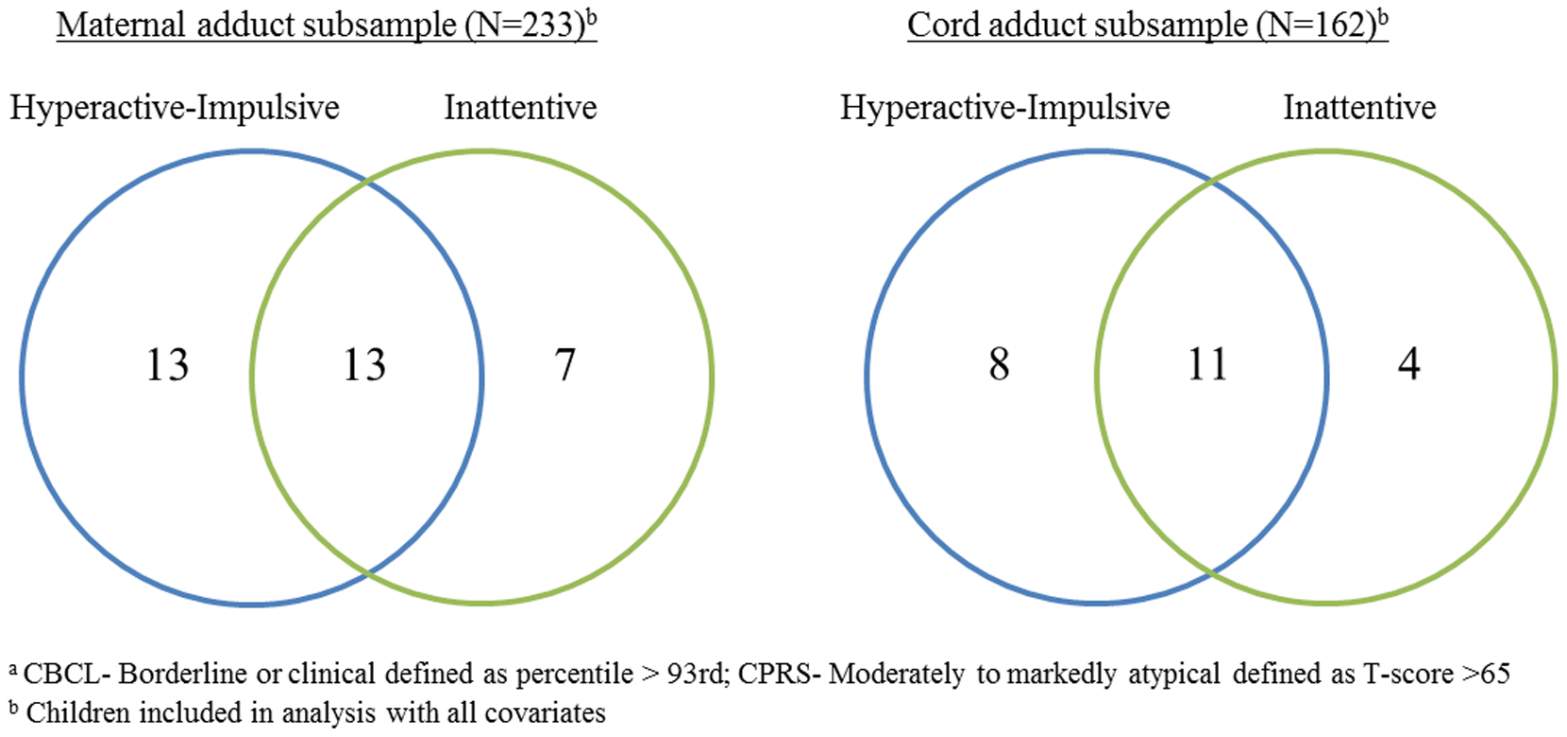
Number of children categorized as moderately to markedly atypical on the CPRS DSM-IV Hyperactive Impulsive and CPRS DSM-IV Inattentive Subscales^a^.

Table 2 summarizes the distribution of CBCL and CPRS scores in the entire sample. Table 3 shows the number of children in the borderline or clinical range on the CBCL, and the number in the moderately to markedly atypical range on the CPRS. Consistent with other studies, there was substantial overlap between the number of children categorized in the moderately to markedly atypical range on the Hyperactive-Impulsive and Inattentive problems, as shown in Figure 1.

**Table 2:** Distribution of CBCL and CPRS Scores in children at age 9 (N = 250^a^).

	Score Range		Mean of Scores		Percent in Borderline or clinical or Moderately to markedly atypical range^b^
Outcomes	T Score	Raw Score	T score	Raw score	
CBCL DSM-oriented Attention Deficit/Hyperactivity Problems	50–80	0–14	53.9	3.1	7.2
CPRS subscales					
ADHD Index	40–89	0–34	49.8	6.4	8.4
DSM-IV Total	40–90	0–48	50.8	9.2	10.4
DSM-IV Hyperactive- Impulsive	41–90	0–25	52.8	4.7	10.8
DSM-IV Inattentive	40–88	0–25	48.9	4.5	8.4

a Children included in analysis with all covariates.

b CBCL “borderline or clinical” defined as percentile ≥93^rd^; CPRS “moderately to markedly atypical” defined as T-score >65.

**Table 3:** Number of children scoring in the borderline or clinical range on the CBCL and in the moderately to markedly atypical range on the CPRS in the analyses with maternal adducts or cord adducts^a^.

	Maternal Adducts (N = 233)	Cord Adducts (N = 162)
CBCL DSM-oriented Attention Deficit/Hyperactivity Problems (Borderline or clinical^b^)	18	15
CPRS subscales (Moderately to markedly atypical^b^)		
ADHD Index	20	14
DSM-IV Total	25	19
DSM-IV Hyperactive Impulsive	26	19
DSM-IV Inattentive	20	15

a Children included in analysis with all covariates.

b CBCL- Borderline or clinical defined as percentile ≥93^rd^; CPRS- Moderately to markedly atypical defined as T-score >65.

Table 4 summarizes the associations between maternal (N = 233) and cord adduct (N = 162) exposure and CBCL DSM-oriented Attention Deficit/Hyperactivity problems and all CPRS outcomes, adjusting for postnatal PAH exposure and selected covariates. When considering outcomes analyzed as continuous raw scores, all CPRS subscales were positively and significantly associated with high maternal adduct exposure. After dichotomizing the outcome measures, those with high maternal adducts had odds of being categorized as moderately to markedly atypical on the DSM-IV Inattentive and DSM-IV Total scales 5.06 (95% CI [1.43, 17.93]) and 3.37 (95% CI [1.10, 10.34]) times greater than those with low maternal adducts. High maternal adduct exposure was also significantly and positively associated with the CDCL DSM-oriented Attention Deficit/Hyperactivity Problems scale, though results did not reach statistical significance.

**Table 4:** Associations between PAH Exposure and CBCL DSM-oriented Attention Deficit/Hyperactivity problems and ADHD Behavior Problems on the CPRS Subscales adjusting for postnatal exposure^a^.

	Maternal Adduct (N = 233)	Cord Adduct (N = 162)	
Outcomes analyzed continuously			
	β_adducts_(95% CI^b^)	β_adducts_ (95% CI^b^)	
CBCL DSM-oriented Attention Deficit/Hyperactivity Problems	0.13 (−0.03, 0.29)	−0.04 (−0.23, 0.15)	
CPRS subscales			
ADHD Index	**0.14 (0.03, 0.25)** *	−0.06 (−0.19, 0.07)	
DSM-IV Total	**0.16 (0.07, 0.26)** *	0.009 (−0.10, 0.12)	
DSM-IV Hyperactive-Impulsive	**0.16 (0.03, 0.29)** *	0.10 (−0.05, 0.26)	
DSM-IV Inattentive	**0.17 (0.04, 0.31)** *	−0.09 (−0.25, 0.06)	
Outcomes analyzed dichotomously c			
	OR^b^ (95% CI^b^)	OR^b^ (95% CI^b^)	
CBCL DSM-oriented Attention Deficit/Hyperactivity Problems	1.48 (0.38, 5.79)	1.17 (0.24, 5.66)	
CPRS subscales			
ADHD Index	1.83 (0.61, 5.54)	0.93 (0.22, 4.01)	
DSM-IV Total	**3.37 (1.10, 10.34)** *	1.70 (0.47, 6.17)	
DSM-IV Hyperactive-Impulsive	1.58 (0.55, 4.52)	1.04 (0.30, 3.61)	
DSM-IV Inattentive	**5.06 (1.43, 17.93)** *	1.32 (0.31, 5.56)	

a Adjusting for postnatal PAH exposure (measured by metabolites at ages 3 or 5, adjusted for specific gravity), prenatal ETS, child sex, maternal education, child ethnicity, gestational age, maternal demoralization, heating season, HOME caretaking environment, maternal intelligence, child age at assessment, maternal ADHD, child anxiety/depression at age 9.

b OR stands for odds Ratio; CI stands for Confidence Interval.

c CBCL- Borderline or clinical defined as percentile ≥93^rd^; CPRS- Moderately to markedly atypical defined as T-score >65.

*p-value <0.05.

In separate models with log transformed adduct and metabolite values as the exposure variables, the direction and significance of the associations between adducts and outcomes were the same as the models with the dichotomized exposure with the exception of the CBCL DSM-oriented attention deficit hyperactivity scale raw score and CPRS DSM-IV Total scale raw score, which became borderline significant (p = 0.06, and p = 0.08, respectively) (data not shown).

Parallel analyses in the smaller number of subjects with available cord adduct data (N = 162) exposure found non-significant or borderline significant associations with all outcomes (Table 4).

## Discussion

The present results suggest that high prenatal exposure, taking into account the potential effects of postnatal PAH exposure, may increase the risk of ADHD behavior problems. ADHD is a disorder that is known to impact school performance, social relationships, and occupational performance [61]–[66]. In the U.S. the annual societal cost of illness for ADHD is estimated to be between $36 and $52 billion, and the annual cost per individual is estimated to be $12,005 to $17,458 (2005) [67], [68].

To our knowledge, there have been no prior epidemiological studies on the role of pre- and post-natal PAH exposure, here measured by chemical-specific biomarkers, on ADHD in school-age children. Prior experimental research and limited epidemiological studies have suggested links between PM and air pollution (elemental carbon and black carbon) and ADHD symptoms [33]–[35]. In the present longitudinal study high maternal adduct levels were not significantly associated with Attention Deficit/Hyperactivity problems on the CBCL screening test, but on the more detailed CPRS, consistently significant associations with a number of ADHD-related outcomes were seen. In particular, significant associations between high maternal adducts and the DSM-IV Total and DSM-IV Inattentive scales were observed in models treating CPRS scores as continuous and dichotomous outcomes. Consistency in the results across both of these outcome measures strengthens the conclusion that inattention is associated with prenatal PAH exposure.

The maternal and cord adducts were significantly but only modestly correlated (r = 0.28, p<0.0001), probably because of the immaturity of the metabolic/detoxification and DNA repair systems in the fetus compared to the adult [69] and the differing genetic profiles of the mother and the child. The stronger relationship between the maternal adducts and ADHD-related outcomes than between the cord adducts and the same outcomes could be attributable to the effects of exposure on placental function and/or the fact that high levels of maternal adducts indicate that the mother has been highly exposed and is an efficient activator of PAH, resulting in higher transplacental exposure to reactive PAH intermediates. We used urinary PAH metabolites to assess postnatal exposure. This biomarker has been employed in many studies as an indicator of PAH exposure in the general population [70]. Although they represent recent exposure [71], the metabolites can provide a chronic measure of ambient PAH in populations with constant exposure [72].

The mechanisms by which PAH exposure might affect the developing brain are not fully understood. Several pathways have been suggested including endocrine disruption [73]–[75], binding to receptors for placental growth factors resulting in decreased exchange of oxygen and nutrients [76], binding to the human Ah receptor to induce P450 enzymes [77], DNA damage resulting in activation of apoptotic pathways [78]–[80], oxidative stress due to inhibition of the brain antioxidant scavenging system [81], and epigenetic alterations [82]. The prenatal period is critical because of the extensive structural and cellular-level changes that occur during this stage of development. However, because brain development and growth occurs throughout childhood, postnatal exposures to environmental pollutants may also affect children's neurodevelopment and behavior [83].

The strengths of the study include our ability to account for a number of potential confounding variables and to draw upon individual pre- and post-natal exposure data from biomarker and questionnaire data. We were able to use two complementary age-appropriate instruments to measure ADHD–related behaviors. The CBCL screens for various childhood behavior problems, including ADHD [53]. The CPRS tests specifically for ADHD and related problem behaviors and is intended to be more diagnostic than the CBCL [54]. Due to the prospective nature of our cohort study we were able to assess the association between environmental exposures sustained prenatally and future development of ADHD-related behaviors in childhood.

A limitation of the study is that unmeasured factors such as other pollutants, stress, and noise may have contributed to residual confounding. In addition, the number of children with moderately to markedly atypical outcomes on the CPRS (cases) was small, resulting in fairly wide confidence intervals around the odds ratios. However, the confidence intervals around the effect estimates for the continuous outcomes are much tighter. Although of interest, we did not have complete data on exposure to lead or mercury and were unable to account for this in our models. We were also unable to evaluate the effects of individual postnatal PAH metabolites that may have differing toxicities; however benzo[a]pyrene is an important, toxic member of the class of PAH. Finally, generalizability was reduced by the ethnicity restriction of our cohort (African-American and Dominican) and our exclusion of active smokers, illicit drug users, and women with pre-existing disease.

## Conclusions

In conclusion, this study provides evidence that early exposure to environmental PAH may contribute to ADHD behavior problems in children. The results require confirmation but are of concern since children with ADHD are at greater risk of risk-taking behaviors [84], poor academic performance [85], and lower earnings in adulthood [86], [87]. ADHD imposes large costs on society, estimated to range between $36 billion and $52 billion annually [67], [68].

PAH are widespread in urban environments worldwide largely as a result of fossil fuel combustion. Fortunately, it is possible to reduce airborne PAH concentrations using currently available pollution controls, greater energy efficiency, the use of alternative energy sources, and regulatory intervention to control polluting sources.
